# Improved cancer risk stratification of isoechoic thyroid nodules to reduce unnecessary biopsies using quantitative ultrasound

**DOI:** 10.3389/fendo.2024.1326188

**Published:** 2024-02-02

**Authors:** Poorani N. Goundan, Theresa Lye, Andrew Markel, Jonathan Mamou, Stephanie L. Lee

**Affiliations:** ^1^ Section of Endocrinology, Diabetes, Nutrition and Metabolism, Boston Medical Center, Chobanian Avedisian School of Medicine, Boston, MA, United States; ^2^ Department of Radiology, Weill Cornell Medicine, New York, NY, United States; ^3^ Topcon Advanced Biomedical Imaging Laboratory, Topcon Healthcare, Oakland, NJ, United States; ^4^ Department of Biomedical Engineering, Tulane University, New Orleans, LA, United States

**Keywords:** thyroid nodule, thyroid cancer, fine needle aspiration, thyroid ultrasonography, thyroid cytology

## Abstract

**Objective:**

Gray-scale ultrasound (US) is the standard-of-care for evaluating thyroid nodules (TNs). However, the performance is better for the identification of hypoechoic malignant TNs (such as classic papillary thyroid cancer) than isoechoic malignant TNs. Quantitative ultrasound (QUS) utilizes information from raw ultrasonic radiofrequency (RF) echo signal to assess properties of tissue microarchitecture. The purpose of this study is to determine if QUS can improve the cancer risk stratification of isoechoic TNs.

**Methods:**

Patients scheduled for TN fine needle biopsy (FNB) were recruited from the Thyroid Health Clinic at Boston Medical Center. B-mode US and RF data (to generate QUS parameters) were collected in 274 TNs (163 isoechoic, 111 hypoechoic). A linear combination of QUS parameters (CQP) was trained and tested for isoechoic [CQP(i)] and hypoechoic [CQP(h)] TNs separately and compared with the performance of conventional B-mode US risk stratification systems.

**Results:**

CQP(i) produced an ROC AUC value of 0.937+/- 0.043 compared to a value of 0.717 +/- 0.145 (p >0.05) for the American College of Radiology Thyroid Imaging, Reporting and Data System (ACR TI-RADS) and 0.589 +/- 0.173 (p >0.05) for the American Thyroid Association (ATA) risk stratification system. In this study, CQP(i) avoids unnecessary FNBs in 73% of TNs compared to 55.8% and 11.8% when using ACR TI-RADS and ATA classification system.

**Conclusion:**

This data supports that a unique QUS-based classifier may be superior to conventional US stratification systems to evaluate isoechoic TNs for cancer and should be explored further in larger studies.

## Introduction

A long-standing concern in the management of thyroid nodules (TNs) is the ineffectiveness of risk stratification of isoechoic TNs as cancer or benign using gray-scale ultrasound (US). The American Thyroid Association (ATA) TN classification system and the American College of Radiology Thyroid Imaging, Reporting and Data System (ACR TI-RADS) use high-risk US features including hypoechogenicity, irregular margins and microcalcification to assign a risk level for malignancy (1, 2). The high-risk features identified in these systems are, however, more specific for the classic papillary thyroid cancer subtype. Isoechoic TNs are very common and are more likely to undergo fine needle biopsy (FNB) due to their larger size (3). While a majority of isoechoic TNs are benign, some malignant TNs (follicular thyroid cancer, follicular variant of papillary thyroid cancer and 20% of classic papillary thyroid cancer) demonstrate an isoechoic appearance on US (4, 5). The current ACR TI-RADS TN classification system would not biopsy and completely miss these isoechoic cancers if partially cystic in appearance. The ATA classification system classifies isoechoic TNs as low suspicion and recommends FNB for a size greater than 1.5 cm regardless of other high-risk features such as hyperechoic foci or invasive margins. Follicular cancer and those that behave similarly have a higher risk for distant metastatic disease compared to papillary thyroid cancer making it important that these TNs undergo FNB appropriately (6). At the same time, considerable health care cost and patient and provider anxiety associated with invasive procedures (i.e., FNB, surgery) for benign disease highlight the need to avoid unnecessary FNBs in benign TNs. Therefore, an imaging technique that uniquely allows analysis of isoechoic TNs to reduce unnecessary invasive FNBs in benign TNs without missing cancer will improve the quality of medical care provided to patients with TNs.

Quantitative US (QUS) is an imaging method that utilizes data from raw ultrasonic radiofrequency (RF) echo signals to assess properties of tissue microarchitecture (7–11). Most of the information contained in RF data is discarded in B-mode gray-scale US imaging that is typically used in clinical care. QUS generates numerical parameters that are a function of the underlying microstructure of the interrogated tissue (e.g., effective scatterer size and effective acoustic concentration) (8, 9). Our group has previously demonstrated the use of this clinically novel US technique in the risk stratification of TNs (12). The area under the receiver operating characteristics (ROC) curve of a linear QUS-based classifier (combination of QUS parameters or CQP) was 0.857 +/- 0.033, and statistically the same as that of ACR TI-RADS and ATA risk classification system for discriminating between malignant and benign TNs (p = 0.327 and p =0.041, respectively) but without the limiting factor of clinician inexperience in thyroid sonography. This CQP classifier also demonstrated a 44 to 66% reduction in unnecessary FNBs which outperformed the reduction using the ACR TI-RADS and ATA risk classification systems with a negative predictive value of 97 to 100%. We now report the outcomes of a preliminary study in which different QUS-based classifiers were created for isoechoic and hypoechoic TNs to determine if cancer-risk stratification improves.

## Materials and methods

The study was performed following institutional review board approval. Details regarding subject recruitment, data collection, RF data processing have been outlined in a prior publication (12). Briefly, patients with one or more TNs who were either undergoing an FNB or had a prior FNB were recruited from the Thyroid Health Clinic at Boston Medical Center. A GE LOGIQ-E9 US scanner (GE Healthcare, Chicago, IL) was used for acquiring RF data utilized for computing QUS parameters using the reference phantom method (13). RF data capture is available natively on the LOGIQ-E9 and therefore no modification of the instrument was necessary. A software key provided by the manufacturer had to be input once to activate RF data capture. TNs with significant cystic area or macrocalcification anterior to the region of interests were not included in the analysis due to interference with US wave propagation. Non-invasive follicular thyroid neoplasms with papillary like features (NIFTPs) were not included due to small numbers in the data set. Investigators who are experienced ultrasonographers reviewed gray-scale US images from the picture and archiving, and communications system (PACS) and determined the echogenicity of TNs. TNs that were designated as isoechoic or hyperechoic were categorized as isoechoic for this study. TNs that were designated as mildly hypoechoic or very hypoechoic were categorized as hypoechoic. A combination of cytology, molecular testing using ThyroSeq genomic classifier (v2 or v3) (CBLPath, Inc., Rye Brook, NY) and surgical pathology was used to classify TNs as benign or cancer. A TN was categorized as benign if it had benign cytology (Bethesda II), or indeterminate cytology (Bethesda III or IV) without any high-risk molecular test result or if surgical pathology did not show any evidence of malignancy. A TN was classified as cancer if found to have Bethesda VI cytology or if surgical pathology demonstrated malignancy. In one subject, a TN with high-risk US features was categorized as malignant based on the presence of a suspicious cervical node that was positive for metastatic thyroid cancer on FNB. Similar methods described in prior publications and in our prior study were used for RF data processing and QUS parameter estimation (7, 12, 14). A combination of QUS parameters were tested using a Fisher linear discriminant approach and classification performance was assessed using ROC curves. Statistical analysis for the study was performed using the MATLAB statistical toolbox (The MathWorks, Inc., Natick, MA).

An optimal linear combination of QUS parameters (CQP) was derived individually for isoechoic TNs [CQP(i)] and hypoechoic TNs [CQP(h)]. The performance of these classifiers was compared to a classifier that was trained using TNs irrespective of echogenicity [CQP(c)] and also to currently used gray-scale TN classification system, ACR TI-RADS and ATA classification system.

## Results

A total of 274 TNs were included in the final analysis. Of these, 163 were categorized as isoechoic (158 benign and 5 cancer) and 111 as hypoechoic (86 benign and 25 cancer) [**Table 1**]. The prevalence of malignancy in TNs categorized as isoechoic was 3.1% and in those categorized as hypoechoic was 22.5%.

**Table 1:** TN characteristics and categorization.

	Isoechoic TN	Hypoechoic TN
No. of nodules	163	111
Average maximal diameter (cm)	2.8 [range 1 - 7]	2.2 [range 0.9 – 7.5]
Final Classification		
Benign, n	158 (96.9%)	86 (77.5%)
Cancer, n	5 (3.1%)	25 (22.5%)
- cPTC	3 (60%)	15 (60%)
- fvPTC	2 (40%)	3 (12%)
- FTC	0	2 (8%)
- Anaplastic thyroid cancer	0	1 (4%)
- Bethesda VI cytology	0	3 (12%)
- Cytology of cervical nodule positive for thyroid malignancy	0	1 (4%)

TN, thyroid nodule; cPTC, classic papillary thyroid cancer; fvPTC, follicular variant papillary thyroid cancer; FTC, follicular thyroid cancer.

(A) Performance of CQP(i) compared to gray-scale US:

The optimal linear combination of QUS parameters (Nakagami shape parameter, intercept, effective scatterer size, and acoustic concentration) for isoechoic TNs – CQP(i) was determined [21.1875 x Avg_NakShapeParam + 2.6668 x Avg_Intercept - 0.89063 x Avg_EffectiveScattererSize - 2.41 x Avg_AcousticConcentration + 25.9495 x NStd_NakShapeParam]. CQP(i) performed with an ROC AUC of 0.937+/- 0.043 [95% CI 0.853 – 1.000] compared to the performance of ACR TI-RADS, 0.717 +/- 0.145 [95% CI 0.436-1.000, p >0.05] and ATA risk stratification system, 0.589 +/- 0.173 [95% CI 0.250 – 0.929, p >0.05] [**Table 2**]. Using the CQP(i) threshold of -61.341 for FNB (i.e., a TN chosen for FNB if the CQP(i) value for the TN is equal or less than the threshold), 119 of 163 (73%) TNs were excluded from FNB with a missed malignancy rate of zero among isoechoic TNs (i.e., all malignant TNs would be selected for FNB). With ACR TI-RADS, FNB would not have been recommended in 91 (55.8%) TNs and one malignant TN would have been missed. With the ATA risk stratification system, FNB could be avoided in 19 (11.7%) TNs, with no missed malignant TN. The reduction in FNB for the ATA system is low in the study as the patient population from which subject recruitment occurred had TNs for which FNB was recommended clinically based primarily on the ATA risk stratification system.

**Table 2:** Comparison of results of individual QUS-based classifiers for isoechoic and hypoechoic TNs compared to ACR TI-RADS and ATA risk stratification system.

	Isoechoic TN (163)			Hypoechoic TN (111)		
	AUC	FNB reduction	Missed cancers, n	AUC	FNB reduction	Missed cancers, n
QUS-based classifier	0.937*	73% (119/163)	0	0.822**	21.6% (24/111)	0
ACR TI-RADS	0.717	55.8% (91/163)	1	0.810	18% (20/111)	1
ATA system	0.589	11.7% (19/163)	0	0.820	1.8% (2/111)	0

TN, thyroid nodule; *based on CQP(i); **based on CQP(h).

When comparing the TNs for which FNB was not recommended, between the CQP(i) and ACR TI-RADS, there was an overlap of 67 TNs in this category. However, 52 TNs for which FNB was not recommended per CQP(i) met criteria for FNB per ACR TI-RADS. Conversely, 23 TNs for which FNB was not recommended per ACR TI-RADS met criteria per the CQP(i) threshold.

The malignant TN missed by the ACR TI-RADS classification system was a 5 cm solid cystic nodule with isoechoic echotexture, with a smooth margin and without any echogenic foci. The surgical pathology of this TN demonstrated a follicular variant papillary thyroid cancer without lymphovascular invasion, extrathyroidal extension or metastatic nodes. Given the size of this TN, it is likely that many clinicians would have chosen to perform an FNB for the TN even if they used ACR TI-RADS system in their clinical practice. When the performance of each TN classification method was revised after removing this nodule, the CQP(i) produced an ROC AUC performance of 0.929 +/- 0.053 [95% CI 0.825-1.000] compared to the performance of ACR TI-RADS of 0.854 +/- 0.095 [95% CI 0.668-1.000] and the performance of ATA risk stratification system of 0.729 +/- 0.149 [95% CI 0.406-1.000].

(B) Performance of CQP(h) compared to gray-scale US:

The optimal linear combination of QUS parameters (acoustic concentration, intercept, midband fit, Nakagami shape parameter, spectral slope) for hypoechoic TNs – CQP(h) was determined [0.062268 x Avg_AcousticConcentration - 0.62066 x Std_Intercept - 0.41233 x Std_MidbandFit - 10.5011 x NStd_NakShapeParam + 0.092736 x NStd_SpectralSlope]. CQP(h) performed with an ROC AUC of 0.822 +/0.051 [95% CI 7.22-0.921], compared to 0.810 +/- 0.049 [95% CI 0.714 – 0.907, p >0.05] for ACR TI-RADS and 0.822 +/- 0.049 [95% CI 0.729 – 0.918, p >0.05] for ATA risk stratification system [**Table 2**]. When CQP(h) threshold of -3.079 to perform an FNB in hypoechoic TNs was chosen, 24 of 86 (21.6%) TNs could be excluded from FNB without missing any malignant TNs. ACR TI-RADS would avoid a FNB in 20 (18%) TNs, and miss one malignant TN (a 1.4 cm hypoechoic TN that was found on surgical pathology to be a follicular variant papillary thyroid cancer without lymphovascular invasion, extrathyroidal extension or metastatic lymph nodes). With the ATA risk stratification system, FNB was avoided in 2 TNs with no missed malignant TNs.

When comparing the TNs for which FNB was not recommended, between the CQP(h) and ACR TI-RADS there was an overlap of 8 TNs in this category. There were 16 TN for which FNB was not recommended per CQP(h) that met criteria for FNB per ACR TI-RADS and there were 11 TNs for which FNB was not recommended per ACR TI-RADS that met criteria per the CQP(h) threshold.

(C) Performance of CQP(i) and CQP(h) compared to a common classifier - CQP(c):

The performance of an optimal linear classifier trained on TNs irrespective of echogenicity, CQP(c) [7.086 x Avg_NakShapeParam – 0.8791x Avg_SpectralSlope + 0.1900 x Avg_AcousticConcentration – 0.6343 x Std_Intercept + 0.1049 x Std_SpectralSlope] was compared to the echogenicity specific classifiers CQP(i) and CQP(h) [**Table 3**]. Using a biopsy threshold of 6.638, the number of TNs excluded from FNB without missing any cancer was 91 (33.2%) when CQP(c) was applied to all TN. Specifically, for isoechoic TNs, 74 (45.4%) FNBs could be avoided which is fewer compared to when CQP(i) was used. When CQP(c) was applied for hypoechoic TNs, 17 (15.3%) FNBs could be avoided.

**Table 3:** Comparison of performance of CQP(c), CQP(i) and CQP(h) in isoechoic and hypoechoic TNs.

FNB criteria	CQP(c)	CQP(c)		CQP(i)	CQP(h)
TN category	All TNs	Isoechoic TNs	Hypoechoic TNs	Isoechoic TNs	Hypoechoic TNs
N	274	163	111	163	111
True Negative, n	91 (33.2%)	74 (45.4%)	17 (15.3%)	119 (73%)	24 (21.6%)
False Positive, n	153 (55.8%)	84 (51.5%)	69 (62.2%)	39 (23.9%)	62 (55.9%)
True positive, n	30 (10.9%)	5 (3.1%)	25 (22.5%)	5 (3.1%)	25 (22.5%)

TN, thyroid nodule.

## Discussion

TNs are made up of a heterogeneous group of histology that includes benign hyperplasia and adenomas, differentiated (papillary and follicular), poorly differentiated, anaplastic and medullary thyroid cancer, thyroid lymphoma and metastatic disease to the thyroid gland (15). All of these subtypes vary in their histological appearance. For example, classic papillary thyroid cancer is characterized by papillary arrangements with a vascular core and psammoma calcification while in follicular thyroid cancer sheets of follicular cells with reduced amounts of colloid are seen with hallmark vascular or capsular invasion. Certain gray-scale US features, such as echogenicity, reflect these differences in the TN architecture. However, while these are more effective in identifying classic papillary thyroid cancers that are hypoechoic and have punctate echogenic foci, it is less the case for the other subtypes.

It is also important to note that we are shifting from an emphasis on TN US to diagnose cancer to a recognition that the management of TNs should also prioritize avoiding unnecessary invasive procedures. Up to 90 to 95% of TNs are benign (16, 17). The incurred cost from FNB, molecular testing and surgery for benign TN is exorbitant (18, 19). In addition, it adds to patient and provider anxiety and increases the risk for post-surgical complications including nerve injury and hypocalcemia. These complications can be reduced, however not eliminated, through various measures such as undergoing surgery by a high-volume thyroid surgeon and use of neuromonitoring devices during surgery.

**Figure 1:**
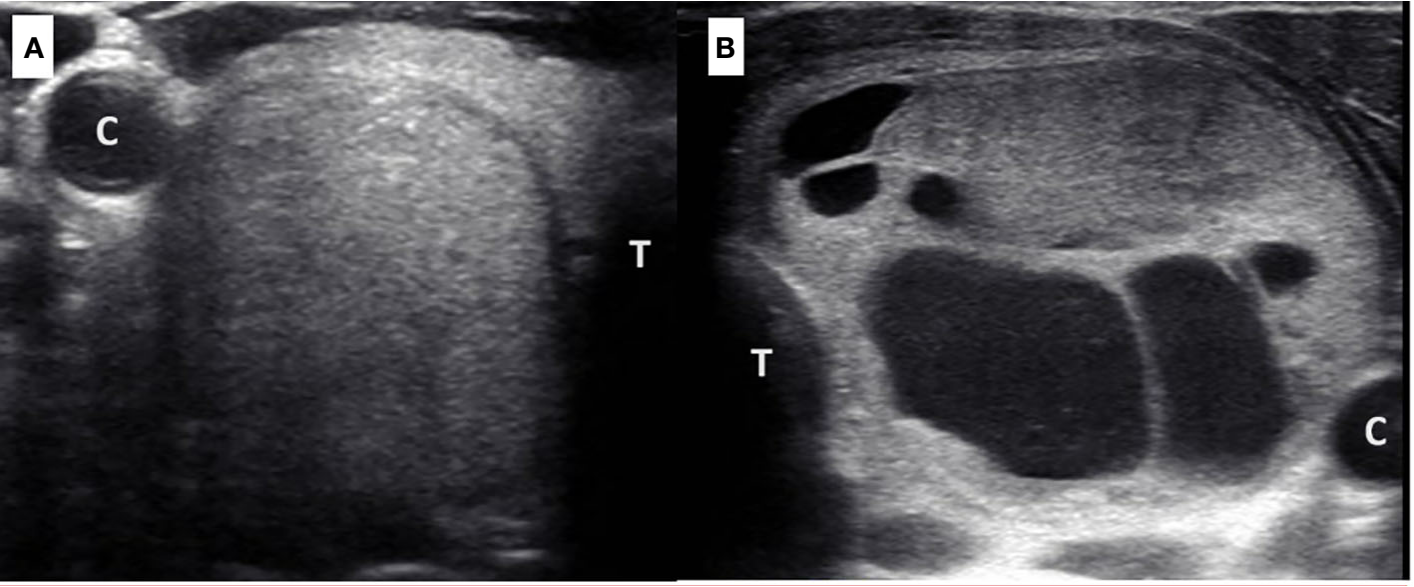
TNs **(A, B)** described with gray-scale US as isoechoic. Nodule **(A)** (right mid 2.6 cm isoechoic TN with well-defined margins, taller-than-wide configuration and no microcalcification; biopsy recommended for TI-RADS 4 if size greater than 1.5 cm) was benign by cytology. TN A had a CQP(i) value of -55.878 (biopsy is not recommended). Nodule **(B)** (left 5.1 cm isoechoic, mixed solid cystic TN with well-defined margins and no microcalcification; biopsy not recommended for TI-RADS 2) was malignant (follicular variant of papillary thyroid cancer) by pathology. TN B had a CQP(i) value of -64.560 measured in the solid region of the nodule (biopsy is recommended). [C, carotid artery; T, trachea].

Creating a separate QUS-based linear classifier for isoechoic and hypoechoic TNs demonstrated improved TN risk stratification, specifically for isoechoic TNs, compared to applying a single classifier for all TNs. The ROC AUC performance of CQP(i) was greater than that of ACR TI-RADS and ATA classification system, but not statistically significantly in the setting of inadequate TN numbers. However, CQP(i) reduces unnecessary FNBs by 73% in isoechoic TNs compared to 55.8% by ACR TI-RADS. **Figure 1** demonstrates two TNs that were both classified as isoechoic. One TN was isoechoic, solid and taller-than-wide and biopsied based on the TI-RADS classification system because the size was >1.5 cm, but was benign by cytology. The second TN was isoechoic and partially cystic and would not have been biopsied based on the TI-RADS classification system. It was found to be a follicular variant of papillary thyroid cancer. These two nodules have very different QUS-based CQP(i) values that would not have recommended a biopsy of the benign isoechoic, solid nodule but would have biopsied the partially cystic isoechoic papillary thyroid cancer. In addition, CQP(i) and CQP(h) together can reduce unnecessary FNB by 52.2% in all TNs without missing a malignant TN. This is improved compared to the reduction in unnecessary FNBs when a single QUS-based classifier is applied to all TNs (45.4%). The relevance of these findings is further highlighted by the fact that 60% of the TNs in the study were isoechoic and 97% of the isoechoic TNs were benign. Of note, while a majority of classic papillary thyroid cancers were hypoechoic, 17% in the study were classified as isoechoic. Follicular thyroid cancers, which are often isoechoic, in the current study were categorized as hypoechoic.

The data demonstrated differences in how the QUS-based classifier and gray-scale US categorized TNs. 43.7% of TNs that did not meet criteria for FNB by the CQP(i) classifier, were recommended for FNB by ACR TI-RADS. 25.3% TNs that did not meet criteria for FNB by ACR TI-RADS were recommended for FNB by the QUS-based classifier. This suggests that the two risk stratification systems are likely assessing different attributes of the TNs and may have a synergistic effect when combined to reduce unnecessary FNBs in TNs. For instance, if one considers combining the CQP(i) and ACR-TI-RADS in the simplest way: a TN is recommended to undergo FNB only if both recommend it, then 142/163 (87%) TNs would have not been recommended for FNB (this includes one malignant TN for which ACR TI-RADS recommend deferring FNB). This demonstrates the tremendous clinical value of combining the microstructural information provided by QUS parameters with the gray-scale US features assessed by an expert ultrasonographer.

Interestingly, the performance of CQP(h), ACR TI-RADS and ATA risk stratification system for hypoechoic TNs was similar. Published literature has demonstrated the ROC AUC performance of gray-scale US in various practice settings ranges from 0.76 to 0.88 (20–23). QUS is not prone to the operator and machine variability seen with gray-scale US, and it can potentially be a useful tool to improve the performance of a less experienced ultrasonographer assessing TNs.

Similar to our prior study, we did not include TNs with a final surgical pathology of NIFTPs due to the low numbers in this preliminary data set. Our institution historically has a low prevalence of NIFTP, which represents 2.3% of all papillary neoplasia (24). In addition, the prevalence of malignancy in isoechoic TNs is low which limits the interpretation of results in our current study. These two concerns can be better addressed in future studies with larger number of subjects. TNs with significant macrocalcification or cystic areas anterior to region of interest in the TN were also excluded because these structures prevent or change the propagation of US RF signal preventing QUS analysis. The authors recognize while TNs were separated into iso- and hypoechoic, these groups are still heterogeneous in their pathology. While echogenicity was chosen in this study to categorize TNs, in future studies the use of other gray-scale US features in combination with QUS should be explored. Secondly, hypoechoic echogenicity can be further categorized as mildly hypoechoic and very or markedly hypoechoic, the latter associated with a higher risk for malignancy (25–27). This needs to be taken into consideration when planning future studies. In this preliminary analysis, the categorization of TNs based on the echogenicity was done manually by the investigators. There can be inter-observer and machine variability. In the future, exploring an objective method for determining echogenicity using either QUS or other techniques should be considered.

For many years we have adhered to a tradition of treating all TNs the same while imaging. Our study is an attempt to apply an algorithmic approach to TN imaging. Our preliminary results are promising and builds compelling case to explore TN imaging keeping heterogeneity in TN histology in mind.

## Data availability statement

The raw data supporting the conclusions of this article will be made available by the authors, without undue reservation.

## Ethics statement

The studies involving humans were approved by Boston Medical Center and Boston University Medical Campus. The studies were conducted in accordance with the local legislation and institutional requirements. The participants provided their written informed consent to participate in this study.

## Author contributions

PG: Formal analysis, Methodology, Writing – original draft, Writing – review & editing. TL: Formal analysis, Methodology, Writing – review & editing. AM: Formal analysis, Writing – review & editing. JM: Formal analysis, Methodology, Writing – review & editing. SL: Formal analysis, Methodology, Writing – review & editing, Writing – original draft.
